# An Increase in Specialist Treatment for Onychomycosis: An Unexplained Tendency. A Retrospective Study of Patients Treated for Onychomycosis in Danish Hospitals from 1994 to 2018

**DOI:** 10.3390/jof9010033

**Published:** 2022-12-24

**Authors:** Pernille Lindsø Andersen, Isabella Friis Jørgensen, Ditte Marie Lindhardt Saunte, Gregor Borut Jemec, Ole Birger Pedersen, Søren Brunak

**Affiliations:** 1 Department of Dermatology, Zealand University Hospital, 4000 Roskilde, Denmark; 2 Department of Clinical Immunology, Zealand University Hospital, 2100 Copenhagen, Denmark; 3 The Novo Nordisk Foundation Center for Protein Research, Faculty of Health and Medical Sciences, University of Copenhagen, 1350 Copenhagen, Denmark; 4 Department of Clinical Medicine, Faculty of Health Science, University of Copenhagen, 1350 Copenhagen, Denmark

**Keywords:** trajectories, onychomycosis, nail fungus, co-morbidities

## Abstract

Onychomycosis is a common disease with a significant negative impact on quality of life. While the disease is usually manageable in general practice, a proportion of patients need specialist treatment in academic hospital clinics. However, it is an unknown question whether the incidence in those needing specialist treatments is changing. Furthermore, the comorbidity burden in this patient population severely affected by onychomycosis has never been characterized. We conducted a retrospective study on patients treated for onychomycosis in Danish hospitals from 1994 to 2018. The cohort was observed for 24 years, and the data comprise 7.2 million Danes and their hospital diagnoses. A disease trajectory algorithm was used to examine the comorbidity burden in the cohort. A total of 2,271 patients received hospital treatment for onychomycosis during the time period, of which 1358 (59.8%) were men. The data show an increase in the incidence of hospital-treated cases since 2012 and that the most common comorbidities in this patient population include cardiovascular disease, alcohol-related diagnoses, and diabetes. One explanation of the increase in specialist treatment may include a general increase in patients with decreased resilience to fungal disease. This lack of resilience may both include an increasing elderly population with atherosclerosis, diabetes, and immunosuppression but also a potential increase in patients treated with immunosuppressive agents. Another possible explanation may include a shift in patient expectations in the case of treatment failure. Thus, patients may have an increasing demand for specialist treatment. While our data document an increase in the number of patients in need of specialist treatment for onychomycosis, we suggest future research to examine the general incidence of onychomycosis but also whether this increase in an apparently recalcitrant disease may be attributed to increased antifungal resistance, more specialist treatment options, or increased attention to dermatomycoses.

## 1. Introduction

Onychomycosis is a common disease with a global prevalence of 4.3% in the general population, and the disease has a significant negative impact in quality of life [1,2,3,4]. It is further believed that the prevalence of onychomycosis is increasing [5]. Usually, onychomycosis is caused by infection, the dermatophytes of which are most commonly *Trichophyton rubrum* [5]. Other pathogens leading to onychomycosis include non-dermatophyte molds and yeasts [6]. Risk factors of onychomycosis comprise advancing age, diabetes, and immunosuppression [6]. Diagnosis and treatment is usually managed by general practitioners (GPs) [6].

The treatment of onychomycosis includes various topical and oral antifungal agents in addition to physical or chemical methods, such as debridement [5,7]. In recent years, other treatment modalities have been suggested and include photodynamic therapies and laser therapy [8]. When deciding on proper treatment it is necessary to take into account the clinical presentation, etiological agent, and the comorbidity status of the patient [6].

As previously stated, onychomycosis is usually manageable in general practice. Yet, a proportion of patients may need specialist treatment in academic hospital clinics [6]. Whether the number of patients in need of such specialized treatment is changing is an unknown question. Furthermore, the comorbidity burden in this population of patients severely affected by onychomycosis has never been characterized.

## 2. Material and Methods

### 2.1. Cohort and Case Definition

The universal Danish healthcare system provides unique possibilities of studying the epidemiology of disease, since each encounter with the healthcare system can be linked to each citizen using the unique personal code provided to each Danish citizen registered in the Civil Personal Register [9]. Thus, to study the disease trajectories of onychomycosis treated in Danish hospitals throughout the entire country, we used registrations in the Danish National Patient Registry (1994–2018) with the diagnosis code B35.1, onychomycosis, in the 10th version of the International Classification of Disease (ICD-10). Since the treatment of onychomycosis in Denmark usually occurs in general practice or by private dermatologists, patients treated in hospitals are those with severe and recalcitrant disease. In Denmark, hospital visits are free of charge and treatment often involves the prescription of topical treatments but may also include physical treatments and surgery. In Denmark, dermatologic hospital departments implies are generally associated with an academic hospital as opposed to private practice dermatologists.

### 2.2. Disease Trajectories

Disease trajectories describe how one disease may precede or follow another disease and are thus not limited to disease progressions. The disease trajectory algorithm has been described elsewhere [10] and is useful for the study of the comorbidities of skin diseases [11,12].

As described above, patients with onychomycosis treated in Danish hospitals were identified with disease pairs (D1 → D2) that occur more frequently in this patient population than expected based on disease prevalence in a matched control group. Relative risks (RR) were calculated to determine the effect size of the disease pair association:

$$RR=\frac{C_{exposed}}{\frac{1}{N}\sum_{i}C_{i}}$$

*C_exposed_* is the number of patients with onychomycosis with disease D1 who develop disease D2. The control group for each patient was defined as 10,000 random patients, but matched by disease D1, age, sex, hospital visit type, and week number of the hospital visit. The control groups were used to assess the prevalence of disease D2. We only included disease pairs with RR > 1 and a temporal directionality tested with binominal tests, which were significant after Bonferroni correction for multiple tests (*p* < 1.21 × 10^−9^).

### 2.3. Statistics

Statistical analyses were performed using Rstudio for Windows (version 1.1.383) and Cytoscape Desktop (version 3.8.2). Descriptives are presented as proportions with frequencies and medians with interquartile range (IQR).

## 3. Results

**Figure 1:**
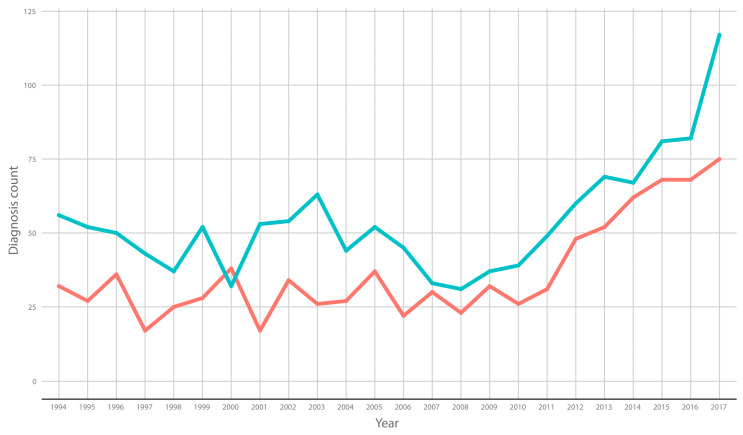
Yearly incidence in patients with onychomycosis treated in Danish hospitals. The blue line illustrates the incidence in men, while the red line illustrates the incidence in women. The available data from 2018 only included the first quarter of the year, and therefore this year’s data have been removed from the graph.

A total of 2271/7191,519 (0.00032%) patients received hospital treatment during the time period, of whom 1358 (59.8%) were men. Of the patients treated for onychomycosis in Danish hospitals, 1425/2271 (62.7%) were registered with onychomycosis as their primary diagnosis, while the rest were admitted to the hospital for reasons other than onychomycosis and were coincidentally treated for onychomycosis. The incidence of patients in need of hospital treatment is presented in Figure 1 and demonstrates that there seems to be an increase in the absolute incidence from 2012 to 2017.

The blue line represents the yearly incidence of men with onychomycosis, while the red line represents the yearly incidence of women with onychomycosis treated in Danish hospitals. Note that as our data from 2018 only included the first quarter of that year, the 2018 data have been excluded from the figure.

**Figure 2:**
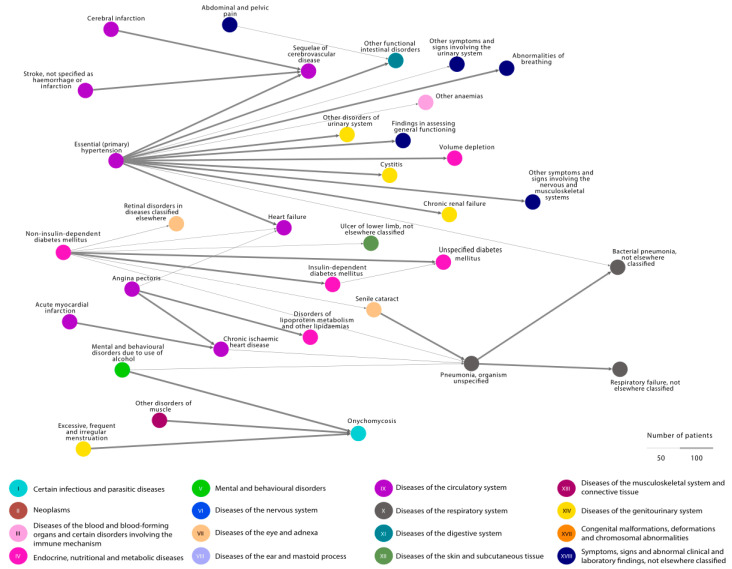
Comorbidity burden and their temporal relations in patients with onychomycosis. All disease pairs illustrated in the figure are overrepresented in patients with onychomycosis compared to controls without onychomycosis. The disease pairs represent a minimum of 50 patients. Thus, the figure illustrates that patients with onychomycosis treated in Danish hospitals often have cardiovascular diseases prior to complications of the first disease, e.g., essential hypertension prior to heart failure and acute myocardial infarction prior to chronic ischemic heart disease prior to pneumonia and respiratory failure. Interestingly, the only diseases occurring in a minimum of 50 patients with onychomycosis are mental disorders due to the consumption of alcohol (green dot); muscle disorders (dark red dot); and excessive, frequent, and irregular menstruation (yellow dot). The different chapters in the 10th version of the International Classification of Disease are illustrated by various colors and Roman numerals.

The illustration of the comorbidities of patients with onychomycosis shows that the comorbidity burden is severe (Figure 2). Comorbidities include cardiovascular disease (purple dots), alcohol-related diagnoses (light green dot), and diabetes (pink dots). Cardiovascular disease includes both the relatively mild diagnosis of essential hypertention but also more severe diseases, such as cerebral infarction, ischeamic heart disease, and heart failure. Diabetes includes both non-insulin-dependent diabetes mellitus, insulin-dependent diabetes mellitus, and diabetes complications, such as retinal disorders. Infections other than onychomycosis, including pneumonia and cystitis, are also overrepresented in this patient cohort.

The thickness of the arrows illustrates the absolute number of patients following the disease trajectory (disease pair). The colors symbolize the different chapters in the 10th version of the International Classification of Disease. A minimum of 50 patients are represented in each disease trajectory. The trajectories illustrated in the network are those overrepresented in patients treated for onychomycosis in Danish hospitals and thus do not necessarily include the onychomycosis diagnosis.

Especially those with a lower age at first hospital onychomycosis diagnosis (30 years of age) have an increased risk of other infections (otitis media) before the onychomycosis diagnosis (supplementary Figure S1). In contrast, those with a higher age at first onychomycosis diagnosis (>50 years of age) recieve alcohol-related diagnoses leading to the onchomycosis diagnosis and present cardiovascular diseases and diabetes and its complications (supplementary Figures S1 and S2).

## 4. Discussion

These data on 2271 patients with onychomycosis treated in Danish hospitals from 1994 to 2018 illustrate that relatively few patients (0.00032%) are referred to specialist treatment in hospitals. Onychomycosis is considered a common disease with a prevalence of 4.9% in Denmark [13], which is comparable with a global population-based prevalence of 4.3% [4]. This is in sharp contrast to the global onychomycosis prevalence of 8.9% in hospital-based cohorts [4]. The higher prevalence may be explained by the fact that most of the included studies were performed at a dermatology departments, which treat patients with onychomycosis but also patients other diseases, such as psoriasis, which are known to be associated with a higher prevalence of onychomycosis [4,14].

This pronounced difference between this study’s onychomycosis prevalence of 0.00032% and the general Danish prevalence of 4.9% only underlines that the majority of onychomycosis cases are treated outside the hospital system in Denmark. Yet, the number of patients referred to specialist treatment seems to have increased during the observation period; especially since 2012. Our data also show an majority of men (59.8%) referred to hospital treatment for onychomycosis compared to women. This sex distribution is in line with the existing literature on the general epidemiology on onychomycosis, although a relative risk of onychomycosis has been suggested to be 2.1 in men compared to women [2,5]. Another explanation for the increasing prevalence during the study period may be the increasing number of inhabitants in Denmark [15] but a growing number of >60-year-old inhabitants may also be a factor, as previous studies have documented a higher prevalence among elderly people [16], with a prevalence of ≥20% in subjects aged ≥ 60 years and ≥50% in those aged ≥ 70 years [17]. The proportion of individuals >60 years of age in Denmark increased by 5% from approximately 15% in 1994 to 25% in 2017, respectively [18,19].

There could be several reasons for the increase in the incidence of patients referred to hospital treatment. One reason may be a general increase in patients with decreased resilience to fungal disease. The severe comorbidity burden observed in those treated for onychomycosis in Danish hospitals possibly illustrates decreased fungal resilience in some individuals. Comorbidities include cardiovascular disease, alcohol-related diagnoses, and diabetes, all of which may result in impaired immunity, as in the case of diabetes, and impaired circulation due to aterosclerosis, which affects the the ability of immune cells to migrate to the periphery of the limbs, as well as nail growth rate, which can increase the risk of recalcitrant infection [20]. These comorbidities are well in line with the general risk factors of onychomycosis [6]. Our data not only show associations with cardiovascular disease and diabetes but also demonstrate that patients with onychomycosis and the aforementioned diseases also suffer from numerous complications related to their cardiovascular diseases and diabetes. One may speculate as to whether patients with onychomycosis refered to hospitals represent a group of patients with more severe cardiovascular disease and diabetes due to the possible impact of these diseases on circulation and thus immunity to the fungal infection of the limbs.

Another reason for the association between onychomycosis and diabetes and its association with aterosclerosis may be that onychomycosis is diagnosed during the hospital-based podiatrist treatment of diabetic patients. The overrepresentation of infections, including cystitis and pneumonia, may illustrate a general immunocompromised state, possibly in elderly individuals. One may also speculate as to whether a general increase in patients treated with immunosuppresive agents, such as methotreaxate, could explain the increase in hospital referrals. Methotrexate increases the risk of dermatophytosis due to its negative effects on lymphocyte activation and proliferation [21]. Patients treated with methotrexate may, however, not be illustrated in our data, as these patients may be treated in general practice or by private dermatologists, e.g., for their psoriasis or rheumatologic disease.

A second reason may be due to the increase in antifungal resistance, observed not only in European countries but also globally [22,23]. In particular, resistance to terbinafine is an emerging problem, as terbinafine is considered the firstline treatment of onychomycosis caused by dermatophytes—the most common etiologic agents [5,23].

A recent recommendation for specialist referral by the European Nail Society concluded that specialist treatment should be performed in cases of “lack of treatment effectiveness, need of additional therapies, concurrent presence of other diseases or comorbidities, severe distal lateral subungual onychomycosis (DLSO), and presence of a dermatophytoma or involvement of the nail matrix” [6]. Our data document an increase in the number of patients referred to hospital-based specialist treatment for onychomycosis. We suggest that this may be due to the severe comorbidity burden in this cohort in line with referral recommendations. This comorbidity burden is in line with the existing literature on the general risk factors of onychomycosis [6]. Additionally, the well-known high relapse rate after medical treatment combined with a high demand for a normal-appearing nails may influence the referral pattern. As new promising treatment modalities of onychomycosis are in the pipeline [8,24,25], one may speculate as to whether this could further increase the number of referrals to specialist treatment.

Our data do not present the incidence of onychomycosis in the general population. Therefore, we suggest future research to examine the yearly incidence of onychomycosis in Denmark to uncover whether the number of referrals to treatment is equivalent to that of the general incidence. We further suggest that future research examines whether this apparent increase in recalcitrant disease may be attributed to increased antifungal resistance, more specialist treatment options, or increased attention to dermatomycoses.

## Data Availability

According to Danish law and regulations, the raw data of this manuscript cannot be shared in public.

## Supplementary Materials

The following supporting information can be downloaded at: https://www.mdpi.com/article/10.3390/jof9010033/s1, Figure S1: Disease pairs, including the onychomycosis diagnosis; Figure S2: Disease pairs overrepresented in patients with the onychomycosis diagnosis.
