# Supplementary material for: An Increase in Specialist Treatment for Onychomycosis: An Unexplained Tendency. A Retrospective Study of Patients Treated for Onychomycosis in Danish Hospitals from 1994 to 2018
Source: J Fungi (Basel). 2022 Dec 24;9(1):33. doi: 10.3390/jof9010033 (PMC9865894; doi:10.3390/jof9010033)
Supplement: Supplementary file 1 [file jof-09-00033-s001.zip › jof-2036679-supplementary.pdf]

Supplementary figures.

Figure S1. Trajectories including the onychomycosis diagnosis.

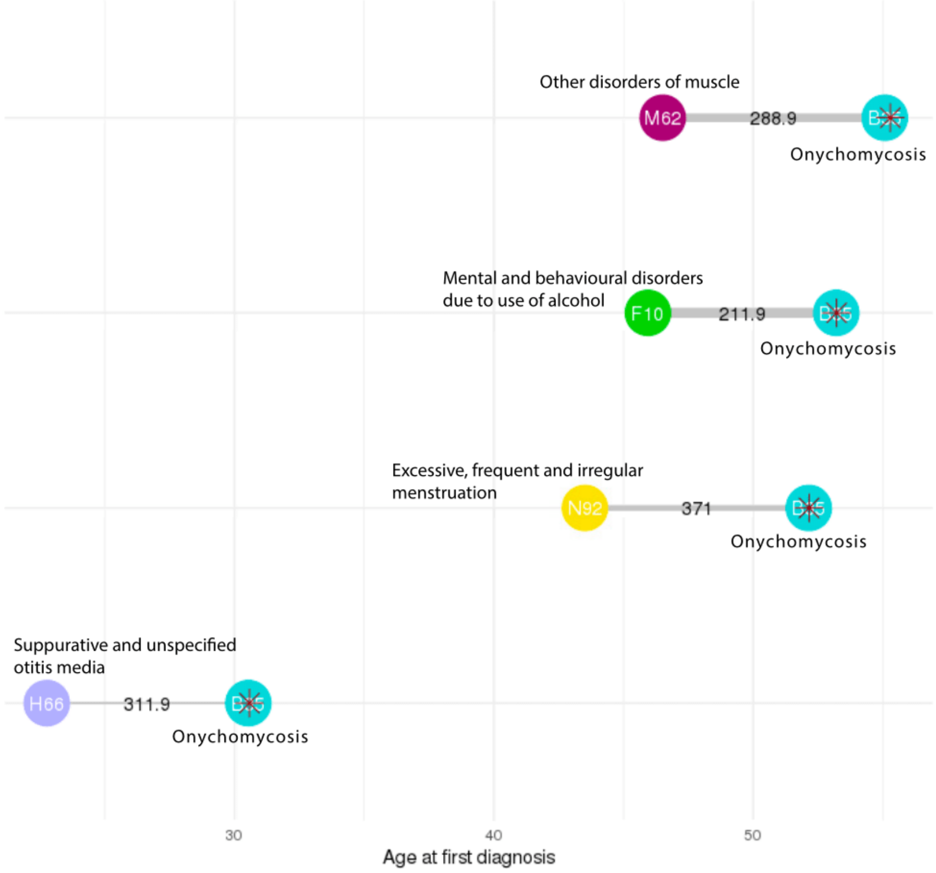

Legend: The numbers indicate the relative risk of following the disease pair, while the width of the lines illustrate the absolute number of patients following the disease pair. The asterisks illustrate the age at first onychomycosis treatment in a Danish hospital. As the occurrence of onychomycosis was very low (0.00032%), the denominator is low when calculating the relative risks, which in turn become correspondingly high as presented in the figure[11]. The number of patients following the trajectories to onychomycosis was 32 from “suppurative and unspecified otitis media”, 118 from “other disorders of muscle”, 136 “mental and behavioural disorders due to use of alcohol”, and 77 from “excessice, frequent and irregular menstruation”.

Figure S2. Disease pairs overrepresented in patients with onychomycosis.

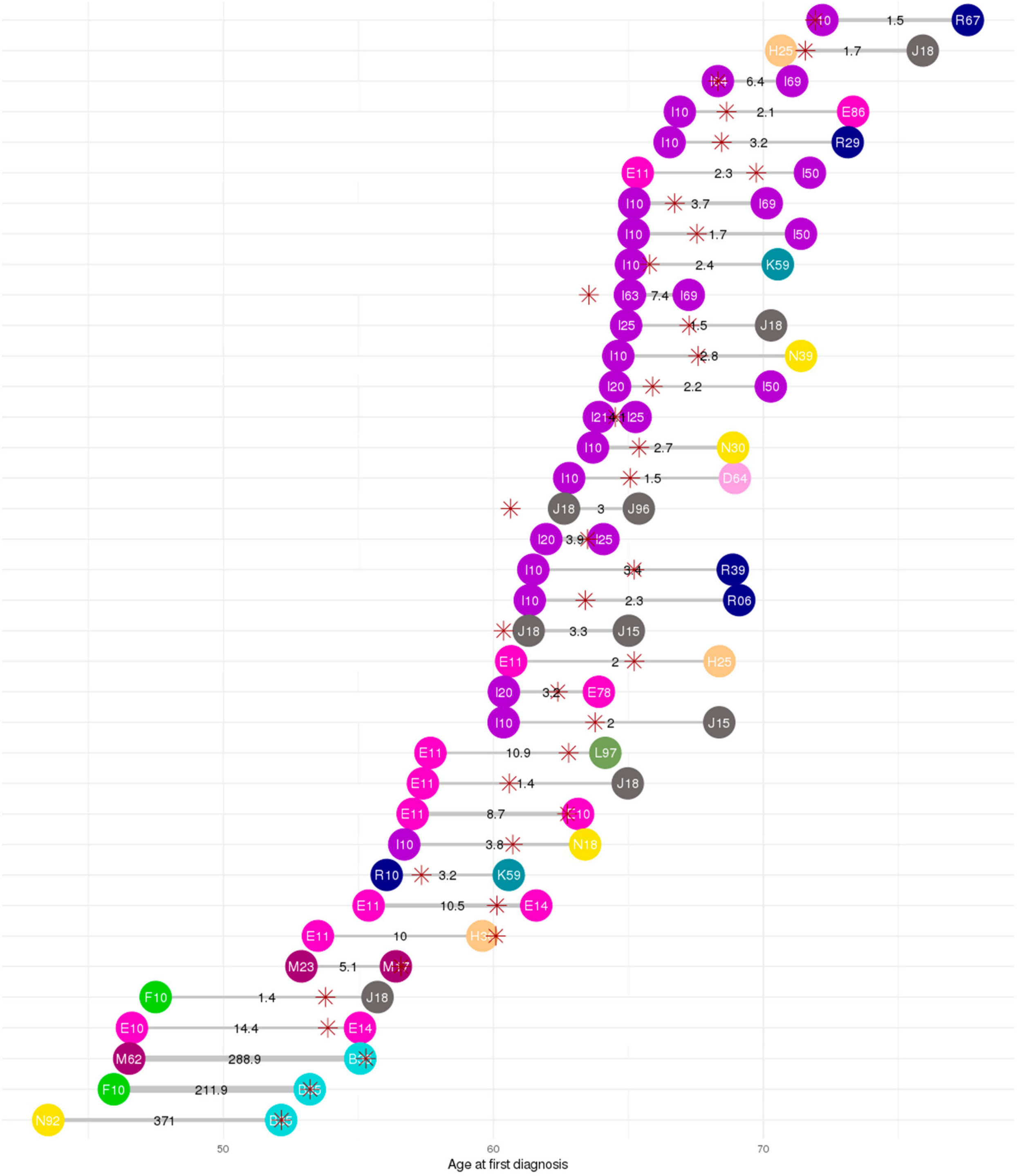

Legend: Min. 50 patients are represented in each disease pair. The numbers indicate the relative risk of following the disease pair, while the width of the lines illustrate the absolute number of patients following the disease pair. The asterisks illustrate the age at first onychomycosis treatment in a Danish hospital.

Light blue) B35: Onychomycosis.

Purple) I10: Essential hypertension. I20: Angina. I21: Myocardial infarction. I25: Ischeamic heart disease. I50: Heart failure. I63: Cerebral infarction. I69: Sequelae of cerebral infarction.

Pink) E10: Type-1 diabetes. E11: Type-2 diabetes. E14: Unspecified diabetes. E78: Hyperlipidemia. E86: Volume depletion.

Light pink) D64: Anemia.

Dark blue) R06: Dyspnea. R10: Abdominal pain. R29: Symptoms and signs involving the nervous and musculoskeletal systems. R39: Symptoms and signs involving the genitourinary system. R67: Reduced ability.

Turquoise) K59: Functional intestinal disorder.

Grey) J15: Pneumonia, bacterial. J18: Pneumonia, unspecified organism. J96: Respiratory failure.

Yellow) N18: Chronic kidney disease. N30: Cystitis. N39: Urinary incontinence. N92: Excessive and frequent menstruation.

Orange) H25: Cataract. H36: Retinal disorder.

Burgundy) M17: Osteoarthritis of the knee. M23: Internal derangement of knee. M62: Unspecified muscle disorder.

Light green) F10: Mental disorders due to alcohol use.
